# Optimization and demonstration of two types of spring-roll dielectric elastomer actuators for minimally invasive surgery

**DOI:** 10.3389/fbioe.2022.1016350

**Published:** 2022-10-04

**Authors:** HaoChen Wang, Saihui Cui, Fuzhou Niu

**Affiliations:** ^1^ School of Mechanical Engineering, Suzhou University of Science and Technology, Suzhou, China; ^2^ Zhongshan Hospital Affiliated to Fudan University, Shanghai, China

**Keywords:** dielectric elastomer actuator, minimally invasive surgery (MIS), design optimization, spring-roll configuration, optimal design method

## Abstract

Resulting from the restricted size of incisions and confined surgical space, the existing rigid and slender minimally invasive surgery (MIS) instruments are inefficient in providing an optimum articulation to handle certain minimally invasive surgery tasks. Thus, developments of novel articulating actuators are of urgent requirement. In this paper, with the aim to enhance the flexibility and maneuverability of surgical instruments in diverse minimally invasive surgery scenarios, two types of spring-roll dielectric elastomer (DE) actuators, namely linear-type and bending-type, are proposed. The actuators’ parameters were optimized and calibrated using a novel step-by-step procedure, based on the characterization and modeling of dielectric elastomer material (VHB 4905). Critical design factors including dimensions of the core spring, the pre-stretch ratio of the dielectric elastomer, and the excitation level of the actuator were identified, while the boundary conditions for the modeling of the actuator were derived from the requirements of minimally invasive surgery applications. The dielectric elastomer actuators’ deformation behavior and force response were analyzed both theoretically and experimentally, and the results from the two approaches were in good agreement. The linear-type actuator could achieve a maximum strain of 29% and a blocking force up to 5.05 N, while the bending-type actuator could achieve angulation over 70° and a blocking force of up to 0.22 N. The proposed actuators are lightweight, compact, and cost-effective, which could provide novel design inspiration for minimally invasive surgery instruments.

## 1 Introduction

Specialized miniature instruments with high-level flexibility and maneuverability can enable the surgeon to perform minimally invasive surgery through tiny incisions and/or natural orifice environments, benefiting in less hospitalization stay and postoperative complication rate compared to traditional surgery. However, the restricted incisions could cause various surgical difficulties including limited surgical operation spaces, lacks of visibility, and constraints on the degrees of freedom (DOFs) of the MIS instruments. In particular, the slender and rigid structure of traditional MIS instruments can only provide a limited range of hardness and accuracy, which can not meet the requirements of precision medical surgical tasks (Vajsbaher et al., 2018). In some common minimally invasive medical procedures, such as single-port laparoscopic surgery (SPLS) or natural orifice endoscopic surgery (NOTES), the whole body of an MIS instrument should work through a narrow single hole (Tei et al., 2021; Hurni et al., 2022), where the surgical field and flexibility of the operation are severely constrained. To deal with the above problems, novel actuators should be designed with adequate mechanical simplicity and robustness to obtain better flexibility and maneuverability.

Dielectric elastomers (DE) are a type of smart materials that could be electrically activated to change their shape. Dielectric elastomers can offer superior performance in terms of energy density and strain capacity compared to other conventional actuation methods (Imboden et al., 2022). Among the DE actuators, acrylic DE actuators are well known for their high strain and mechanical energy density. In particular, the acrylic VHB DE material shows the largest strain up to 380% and actuation pressure up to 7.2 Mpa (Sahu et al., 2022), which enables it a better response and performance surpassing that of natural muscle. Such characteristics make it particularly suitable for applications in robotics, prosthetics, and animatronics (Ji et al., 2019; Li et al., 2019; Di et al., 2021; Guo et al., 2021).

An ideal DE actuator for MIS application should be lightweight, miniature, flexible and safe, while capable of providing sufficient force output for the surgical operation (Le et al., 2016; Runciman et al., 2019). In particular, the outer diameter of the actuator should be less than 10 mm to fit most of the trocar ports. The length of a single actuator should be less than 50 mm to allow at least 3–4 actuators for the articulation of the MIS tools (the usual depth of penetration of MIS tools inside the patient’s abdominal cavity is around 150 mm (Soper et al., 2008)). The blocking force of the bending actuator should be in a range of 0.1–5 N and the linear-type actuator in 5–10 N which are the common operating force in MIS (Golahmadi et al., 2021), while an adequate strain/angulation should also be achieved for the whole articulated system. The power and control unit of the actuator should be moderate for an adequate set-up time and room for surgical operation (Lonner et al., 2021). Accordingly, the design requirements are summarized in Table 1, which are benchmarked against the performances of other actuators which were developed for MIS and already reported in the literature (Moers et al., 2012; Gerboni et al., 2016; Decroly et al., 2020; Wang and El Wahed, 2020; Wang et al., 2020; Decroly et al., 2021; Zhang et al., 2021).

**TABLE 1:** Performance of different actuators and design requirements of DE actuators proposed for MIS applications.

Actuator type	Total length (mm)	Outer diameter (mm)	Strain/Angulation	Blocking force (N)	Power &control unit
Bending-Tendon Driven (Zhang et al., 2021)	48.8	7	180°	0.96∼5	N/A
Bending-Electromagnetic (Wang and El Wahed, 2020)	31.6	10	30°	1.42	Moderate
Bending-Hybird (Wang et al., 2020)	40	11	180°	N/A	Moderate
Bending-Pneumatic Driven (Decroly et al., 2021)	50	6	180°	0.1	Moderate
Bending-Fluidic Driven (Decroly et al., 2020)	17∼43	5	270°	0.04	Small
Linear-Fluidic Driven (Gerboni et al., 2016)	4	9	50%	5.6	Small
Linear-Fluidic McKibben (Moers et al., 2012)	62	1.5	15%	6	Small
Design requirements	30∼50	5∼10	30% (linear) 60° (bending)	5–10 (linear)0.1–2 (bending)	Moderate

**FIGURE 1:**
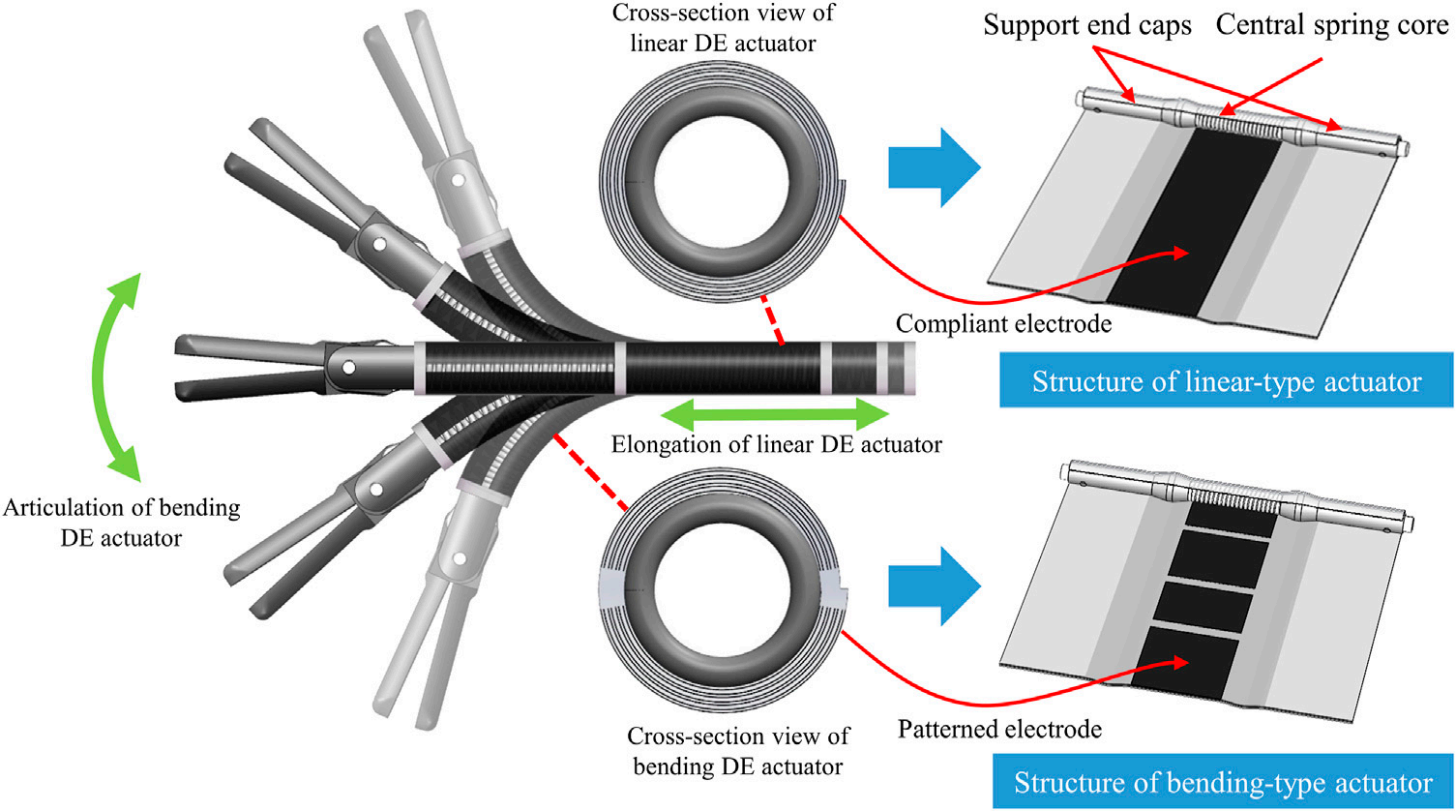
Design concept of linear- and bending-type DE actuators for MIS application.

To meet the above requirements, two types of spring-roll configuration DE actuators were designed in this study. As shown in Figure 1, when the coated area of the electrode was subjected to an external electric field, the DE roll of the linear-type spring-roll DE actuator could reduce its thickness in a circumferential direction and hence produce a linear elongation in its axial direction. Alternatively, the coated area of the electrode could be patterned (segregated) to permit a partial activation of the DE and hence produce a bending action of the actuator. The spring-roll DE actuator was designed to replace part of the rigid stem of conventional MIS tools. By aligning multiple linear- or bending-type spring-roll DE actuators, a flexible MIS instrument could be achieved as illustrated in Figure 1.

Even though DE actuators have been proven to offer large axial and bending actuation, the trade-off between their technical capabilities and the design requirements of the targeted devices has always been a major challenge that impedes their practical implementation. Therefore, the optimal design of the spring-roll DE actuator is critical to validate its feasibility and achieve its ultimate MIS applications. Besides, modeling of the DE actuator remains difficult due to the inherent nonlinear hyperelasticity of the DE material. Moreover, performance demonstrations needed to be conducted for verifying its stability and feasibility for articulating the MIS instruments.

In this paper, the three most important design factors that are expected to critically affect the overall performance of the developed actuator were identified, including the dimensions of the core spring, the pre-stretch ratio of the DE in different directions, and the excitation level of the actuator. Based on the characterization and modeling of the DE and the spring, a novel step-by-step optimization process of the above parameters was developed to address the critical trade-off between the technical capability of the spring-roll DE actuators and the design requirements of MIS applications. In addition, this paper also presents robust design and fabrication techniques for linear and bending-type spring-roll DE actuators. The performance of the prototypes was tested in terms of actuation strain and force, and the experimental results show that the developed DE actuator was promising for MIS uses.

The remainder of this paper is organized as follows. Section 2 addresses the optimal design of DE actuators aiming for MIS applications. Section 3 presents the fabrication process and experimental arrangements. Section 4 gives the results and discussions. And, Section 5 gives the conclusions.

## 2 Optimal design of dielectric elastomer actuators

### 2.1 Optimal design strategies

**FIGURE 2:**
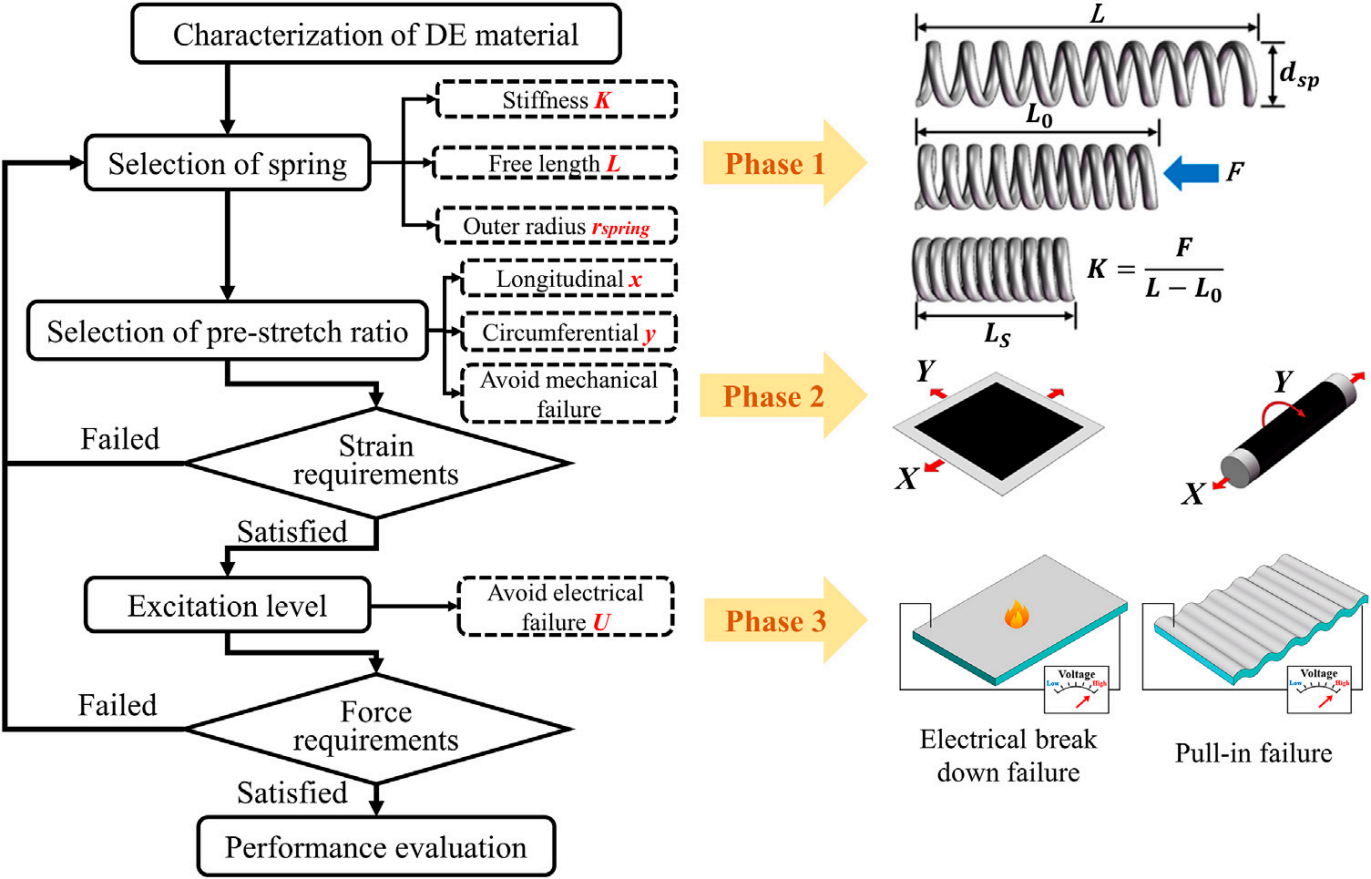
Optimization of the DE actuator for MIS.

In this study, we use boundary conditions derived from the requirements of MIS applications (Table 1) to define the allowable strain and force outputs of the actuators for an enhanced theoretical modeling compared to the previous study (Li et al., 2019; Guo et al., 2021; Prechtl et al., 2021). Accordingly, several design parameters which are expected to critically affect the performance of the developed actuator were identified and targeted as shown in Figure 2.

When the spring-roll DE actuator reached a steady status, the elastic stress of the elastomer in the axial direction was balanced with the sum of two stresses, which are the Maxwell stress induced by the external electric excitation and the operating stress provided by the compressed spring, respectively. The Maxwell stress and the operating stress facilitate the expansion of the DE-film whilst the elastic stress impedes such variation as shown below:

$$\sigma_{elastomer}\left(x,y,\varepsilon \right)=\sigma_{spring}\left(K,L,r_{spring},\varepsilon \right)+\sigma_{Maxwell}\left(U,\varepsilon \right)$$ (1)

Where *x* and *y* are the pre-stretch ratios in the *X* and *Y* direction respectively; *ε* is the strain of the actuator in the longitudinal direction; *K* is the stiffness of the spring; *L* is the free length of the spring; *r*_*spring*_ is the outer radii of the spring; *U* is the applied voltage on the DE.

Accordingly, the optimal design process is mainly divided into three phases, which are corresponded to the optimization of the spring, DE and electrical excitation, respectively. Since the parameters of spring mainly determine the overall size and capabilities of the actuator, it was analyzed in the first place. Subsequently, the design parameters of DE were discussed as they dictate the actuation state of the actuator. Finally, the limitation of electrical excitation level was determined experimentally. As presented in Figure 2, the proposed optimization was progressed iteratively to both unfold the complex interaction between the three phases and maximize the strain/force performance of the developed actuator.

The optimization of the bending actuator is similar to the linear actuator, where the major difference is the pattern of the electrode. In this study, the bending actuator is designed as two degrees of freedom actuator as shown in Figure 1 and its analytical model could be derived from the modeling of the linear actuator (Li et al., 2019). Therefore, the optimization process would stress on the linear spring-roll actuator for simplicity. Accordingly, the discussion of those parameters has progressed step by step in the following sections.

### 2.2 Characterization of dielectric elastomer material

**FIGURE 3:**
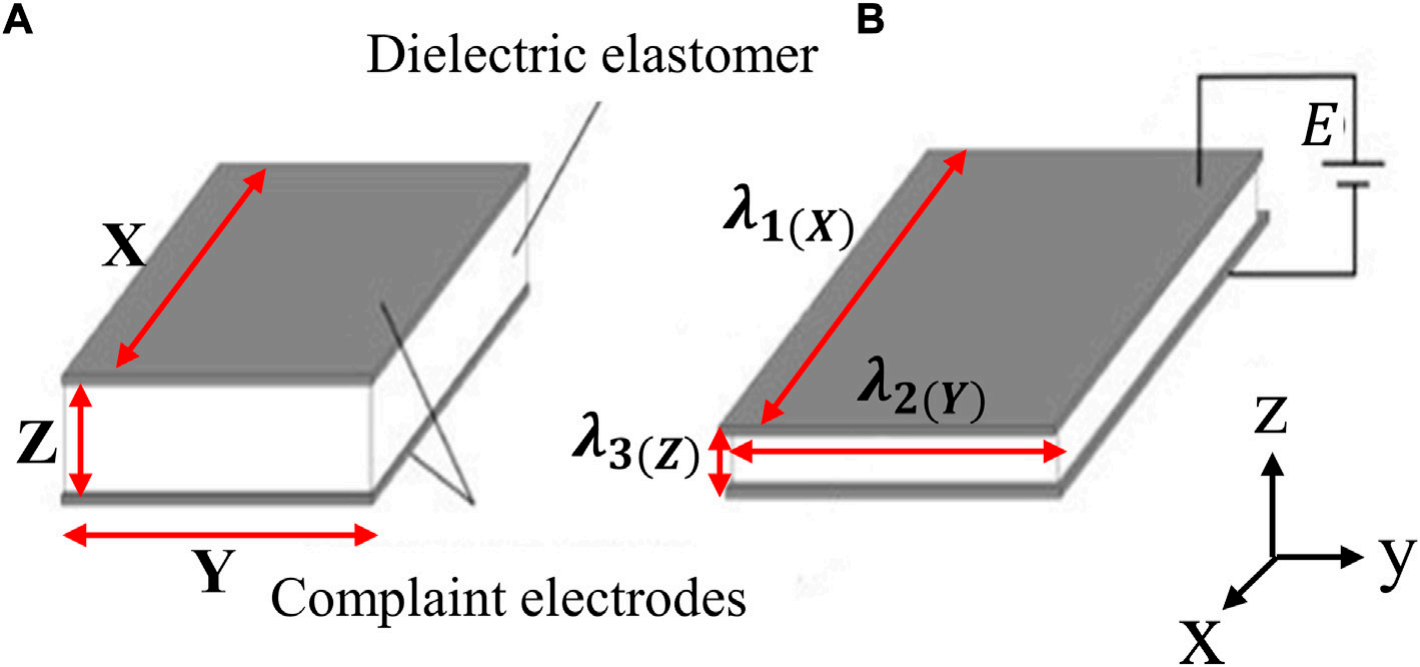
Dielectric elastomer working principle: reference state **(A)** and activated/deformed state **(B)**.

Dielectric elastomers are usually comprised of a thin layer of dielectric film with low in-plane stiffness, which is coated with compliant electrodes on both sides to form a sandwich-like structure. When an electric excitation (usually in the range of several kV) is applied across the compliant electrodes, electrostatic stress, also known as the Maxwell stress, is generated as a result of the attraction of the opposite charges induced on the two sides of the dielectric film. The electrostatic stress would squeeze the dielectric film and cause a deformation in a direction that is normal to the direction of the applied field (Prechtl et al., 2021), as illustrated in Figure 3.

Attributed to the high strain capability and dielectric constant provided by the 3M VHB 4910 film, it has been routinely used for the construction of DE actuators (Li et al., 2019; Guo et al., 2021). However, since the reduction of elastomer thickness could enlarge the outputs of the DE actuator (Li et al., 2019; Guo et al., 2021; Prechtl et al., 2021), 3M VHB 4905 acrylic film (0.5 mm thickness) was employed as the dielectric elastomer instead of 3M VHB 4910 (1.0 mm thickness) in this investigation. Therefore, the characterization of the VHB 4905 film was required. Reports have indicated that the Yeoh model (hyperelastic model) was deemed to be adequate to describe the deformation and stress behavior of 3M acrylic film (Garcia and Trindade, 2019), which are given by:

$$I_{1}=\lambda_{1}^{2}+\lambda_{2}^{2}+\lambda_{3}^{2}$$ (2)

$$\frac{\partial W}{\partial I_{1}}=C_{10}+2C_{20}\left(I_{1}-3\right)+3C_{30}{\left(I_{1}-3\right)}^{2}$$ (3)

$$\sigma_{i}=\lambda_{i}\frac{\partial W}{\partial \lambda_{i}}-P_{s}=\lambda_{i}\frac{\partial I_{1}}{\partial \lambda_{i}}\frac{\partial W}{\partial I_{1}}-P_{s}$$ (4)

where $C_{10}$ , $C_{20}$ and $C_{30}$ are the material constants, *Ps* is the hydrostatic pressure which depends on the boundary conditions of the elastomer, $\lambda_{1}$ , $\lambda_{2}$ and $\lambda_{3}$ are the stretch ratios in the X, Y, and Z directions, respectively which compose a Cartesian coordinate system as illustrated in Figure 3. The X, Y, and Z directions are identified as the longitudinal (axial direction of the DE roll), latitude (circumferential direction of the DE roll), and thickness directions of the DE elastomers.

To characterize the VHB 4905 film in the Yeoh model, the required material parameters were obtained from a set of uniaxial tensile tests which were performed on this film using a Tinius-Olsen tensile machine, model H5KS. This enabled the three main constants $C_{10}$ , $C_{20}$ and $C_{30}$ of the Yeoh model to be estimated for the VHB 4905 elastomer film, which were found to be 14,070, −184.93, and 4.042, respectively.

The viscoelastic behavior of the VHB 4905 material was characterized by a relaxation test with stretch ratio inputs varied between 100% and 400%. The result indicates that the stress relaxation of the film becomes slower after 60 s as it only drops further 3% (to 97%), after 200 s. Therefore, it was decided in this study that the deformed shape of the spring-roll DE actuator is recorded after approximately 60 s from the voltage application, during which the DE actuator is assumed to be fully relaxed.

### 2.3 Selection of spring

The maximum allowable strain (*ε*_max_) of the actuator in the axial direction is a prior parameter that directly determines the capability of linear extension and bending angle for the spring-roll DE actuator. The core spring supports the structure of the activated DE rolls and defines the boundary conditions of the elastomer film (as presented later in sub section 4.2, Figure 12). Any stroke above the maximum compressed length will result in an undesired extension of the compression spring and meet the failure of the actuator. Therefore, the operating stress of spring (*σ*_*spring*_) and maximum allowable strain (*ε*_max_) could be expressed as shown below:

$$\sigma_{spring}=\sigma_{0}-\frac{\varepsilon L_{0}K}{\pi \left(r_{actuator}^{2}-r_{spring}^{2}\right)}$$ (5)

$$\varepsilon_{\max}=\frac{L}{L_{0}}-1=\frac{L}{L-\frac{\sigma_{0}A}{K}}-1=\frac{1}{\frac{KL}{\sigma_{0}\pi \left(r_{actautor}^{2}-r_{spring}^{2}\right)}-1}$$ (6)

Where *L*_*0*_ is the compressed length of the spring in deactivated status; *A* is the cross-section area of the DE stack rolls in the longitudinal direction; *σ*_*o*_ is the initial operating stress provided by the compressed spring acting on the DE rolls; *r*_*actuator*_ is the outer radii of the deactivated DE actuator.

To maximize *σ*_*spring*_ and *ε*_max_, a spring with low free length(*L*), stiffness(*K*), and outer radius (*r*_*spring*_) should be targeted. Also, a larger *r*_*actuator*_ is favored which a maximum value is 5 mm in this design. However, during the fabrication of the prototypes, it was found that if the thickness of the elastomer rolls in the radial direction is larger than the radius of the core spring (*r*_*spring*_ too small), the shape of the actuator would be easily twisted by the elastomers and result in failure of the design. Besides, *L*_*0*_ must be greater than or equal to the solid length (*L*_*s*_) of the spring to enable the stress balance of the actuator and avoid corresponding mechanical failure. In addition, *ε*_max_ should exceed 30% according to Table 1, which gives the following boundary conditions:

$$\left\{ \begin{array}{l} L_{0}=L-\frac{\sigma_{0}\pi \left(r_{actautor}^{2}-r_{spring}^{2}\right)}{K}\geq L_{s}\\ \varepsilon_{\max}=\frac{\sigma_{0}\pi \left(r_{actautor}^{2}-r_{spring}^{2}\right)}{KL-\sigma_{0}\pi \left(r_{actautor}^{2}-r_{spring}^{2}\right)}>30\% \end{array}\right.$$ (7)

According to Eqs. 5, 6, a promotion on *σ*_*o*_ could also increase *σ*_*spring*_ and *ε*_max_, resulting in a better strain performance of the DE actuator. Yet, *σ*_*o*_ equals to the initial elastic stress of the DE film, which depends on the pre-stretch ratio of the elastomer. Finally, a commercialized spring manufactured by Entex Spring company (model 3,364) was selected and the detailed parameters of the spring are given in Table 2.

**TABLE 2:** Parameters of the selected commercialized spring.

Model	Outer diameter (mm)	Inner diameter (mm)	Free length (mm)	Solid length (mm)	Stiffness (N/mm)
3,364	6.35	5.23	63.5	19.89	0.17

### 2.4 Selection of pre-stretch ratio

The effect of the pre-stretch process on the enhancement of the performance of dielectric elastomer has been proven to be significant by several research groups (Li et al., 2019; Guo et al., 2021; Prechtl et al., 2021). Such a process could reduce the thickness of single DE layers before the fabrication of the DE actuator, and hence enlarge the electric field and the corresponding Maxwell stress under the same applied voltage level. The DE could be biaxially pre-stretched in two directions, which are the X (longitudinal) and Y (circumferential) directions, respectively.

**FIGURE 4:**
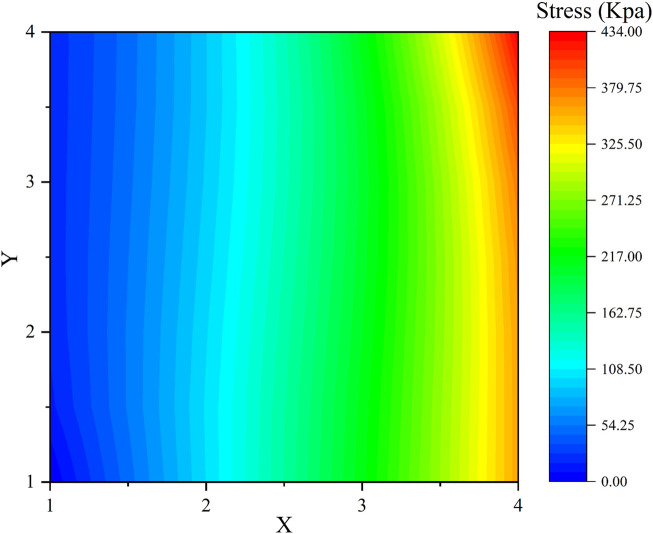
Elastic stress of VHB 4905 in the longitudinal direction with different Pre-stretch ratios.

During the experiments, it was found that the elastomer becomes extremely sensitive and vulnerable when the pre-stretch ratio exceeds four in each direction. Hence the maximum pre-stretch ratio in the *X* and *Y* direction was determined to be four to avoid any mechanical failure. According to Eqs. 2–4, the elastic stress of VHB 4905 in the longitudinal direction with different pre-stretch ratios was displayed in Figure 4.

**FIGURE 5:**
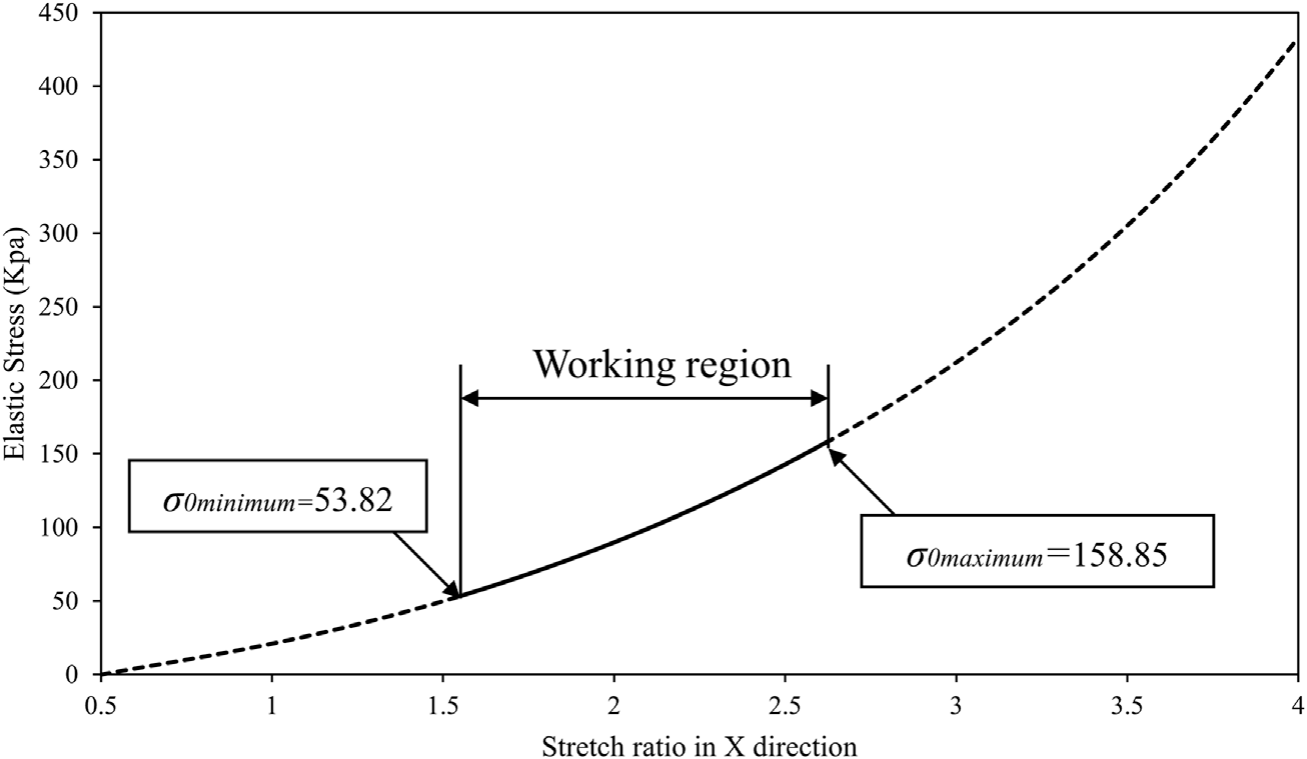
Elastic stress of the DE film in the longitudinal direction with *x* from one to four and *y* as four against the constrain of spring model 3,364.

The elastomer film pre-stretching process aims to increase the Maxwell stress as much as possible by reducing the DE film thickness, but at the same time to keep the elastic stress low in the longitudinal direction. It can be seen from Figure 4 that the pre-stretch ratio in the *Y* direction has little effect on the elastic stress in the *X* direction. Therefore, by considering both maximizing the pre-stretch ratio as well as lower the corresponding elastic stress, a pre-stretch ratio of four was selected in the *Y* direction. In contrast, the selection of pre-stretch ration in the *X* direction should allow the initial elastic stress to fulfill the conditions of Eq. 7. Accordingly, the elastic stress of the DE film in the longitudinal direction with *x* from one to four and *y* as four is displayed in Figure 5.

As presented in the above figure, the allowable pre-stretch ratio *x* under the constraint of spring model 3,364 is between 1.54 and 2.63 when the pre-stretch ratio y is 4. Therefore, and to be on the safe side particularly when high voltages are involved, the pre-stretch ratio *x* was eventually chosen as 2.5.

### 2.5 Excitation level and electrical failure

According to the previous work on DE actuators (Li et al., 2019; Prechtl et al., 2021), the electrostatic pressure (Maxwell stress) acting along the thickness of the dielectric film could be estimated by the equation below:

$$\sigma_{Maxwell}=f_{0}f_{r}{\left(\frac{\varepsilon U}{d}\right)}^{2}$$ (8)

**FIGURE 6:**
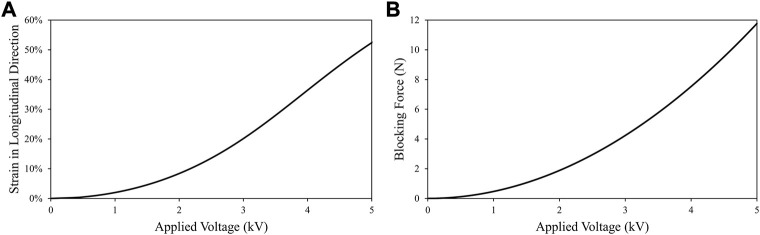
Performance estimation of the linear DE actuator with different excitation levels: **(A)** Strain *versus* applied voltage; **(B)** blocking force output *versus* applied voltage.

Where *f*_0_ is the free-space permittivity (8.85 × 10^–12^ F/m), *f*_r_ is the dielectric constant of the elastomer film, and *d* is the thickness of the DE in deactivated status, *U* is the excitation level (voltage) of the compliant electrode. Accordingly, the Maxwell stress could be enlarged with the increase of the excitation level. By substituting Eqs. 4, 5 and Eq. 8 into Eq. 1, the strain and force output of the developed linear DE actuator *versus* the applied voltage could be estimated as presented in Figure 6.

By considering the design requirements prescribed in Table 1, it could be seen from the above figure that the maximum excitation level of the DE actuator should be above 3.5 kV to achieve a strain of 30% and force output over 5 N. Yet, electrical breakdown failure occurs when the electric field applied across the dielectric elastomer film exceeds the breakdown strength limit of the material (*U* exceeds the limits), which causes an electrical discharge (spark) across the film and hence limits the maximum excitation level of the DE actuator. This type of failure is fatal as the generated heat would dramatically build up in that region between the compliant electrodes, which then causes the film material to burn and be damaged beyond any possible repair. Many factors could affect the breakdown limits of the DE actuator and controversy remains on the estimation of this parameter in literature (Di et al., 2021; Guo et al., 2021). Therefore, in this study, the maximum excitation level of the DE actuator was investigated experimentally.

**FIGURE 7:**
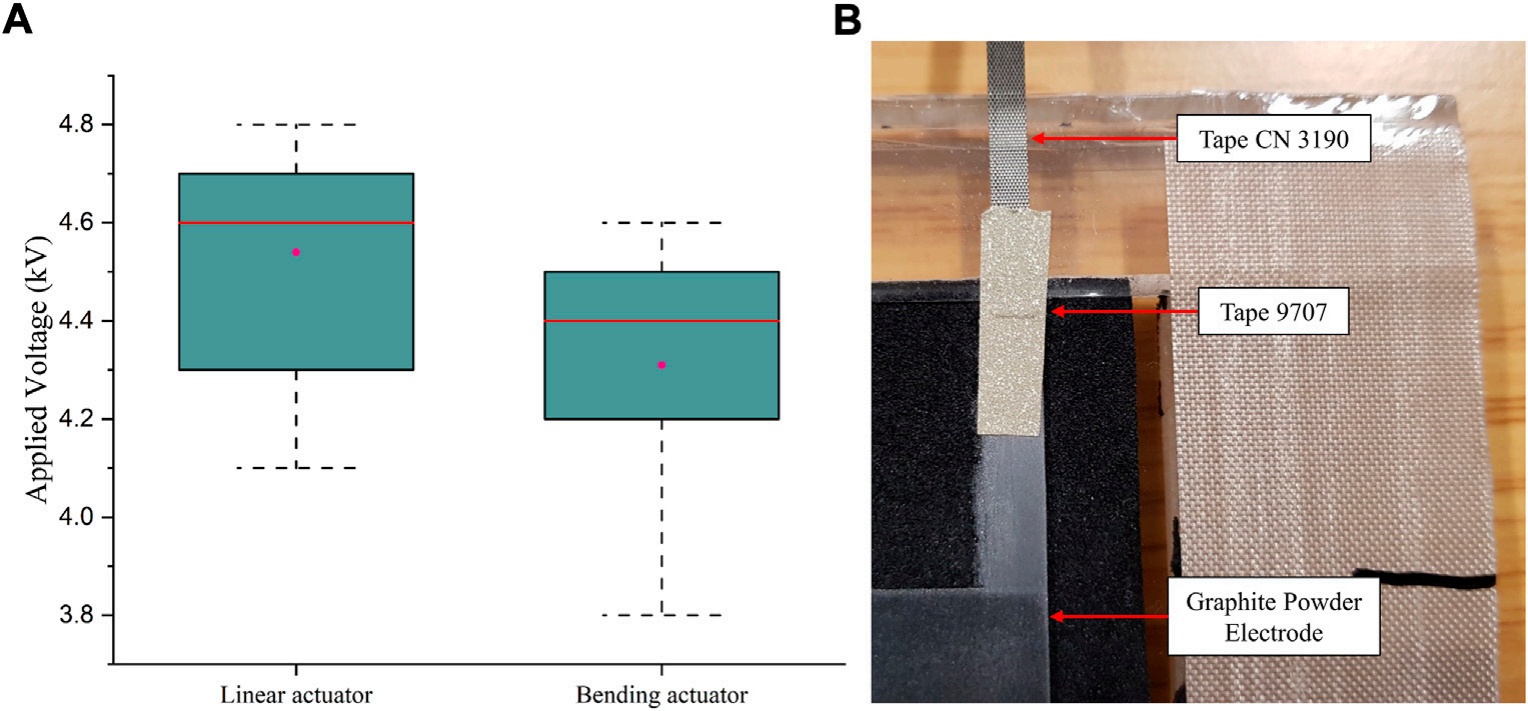
Electrical breakdown failure of the DE actuator: **(A)** Electrical breakdown failure of the bending and linear spring-roll DE actuator; **(B)** Electrical connection area of the dielectric film.

Twenty trials were conducted for linear and bending DE actuators (each with ten trails) and the results are displayed in Figure 7. Electrical conductive adhesive transfer tape 9,707 (thickness 0.05 mm) and tape CN3190 (thickness 0.125 mm) from 3M company were used to facilitate the electrical connection of the DE actuator. However, as a result of the non-uniformity of this area, as shown in Figure 7B, the electrical breakdown failure usually occurs when the applied voltage is above approximately 4 kV. Therefore, in consideration of both the safety during the activation and the design requirements, the maximum excitation level was set as 3.5 kV in this study.

Since the spring was compressed during the activation, the Maxwell stress will always be lower than the elastic stress of the DE, and hence the pull-in failure (Plante and Dubowsky, 2006) (Maxwell stress becomes greater than the elastic stress of the DE) could be dismissed. In summary, the design parameters of the optimized spring-roll DE actuator are listed in Table 3.

**TABLE 3:** Design parameters of the optimized spring-roll DE actuator.

Dielectric Elastomer	VHB 4905
Selected spring	Model 3,364 (Entex Spring)
Pre-stretch ratios	*x* = 2.5 and *y* = 4
Outer diameter	l0 mm
Length	∼24.2 mm
Maximum excitation level	3.5 kV

## 3 Fabrication and experimental arrangement

### 3.1 Fabrication of the dielectric elastomer actuator

An ideal compliant electrode of DE actuator should be able to maintain a high electrical conductivity and good stability at large actuator strain and have negligible in-plane stiffness and thickness. In this study, ALDRICH graphite powder (particle size less than 20 µm) was chosen as the applied electrode since it could be easily smeared on the designated electrode area of the adhesive surface of VHB 4905. Any extra powder material could be blown off from the sticky film surface, and the uniformity of the thickness of the electrode could be guaranteed. To increase its conductivity under large strain level deformations, this electrode material was painted when the DE film was overstretched, which enhanced the maximum strain of the activated area.

**FIGURE 8:**
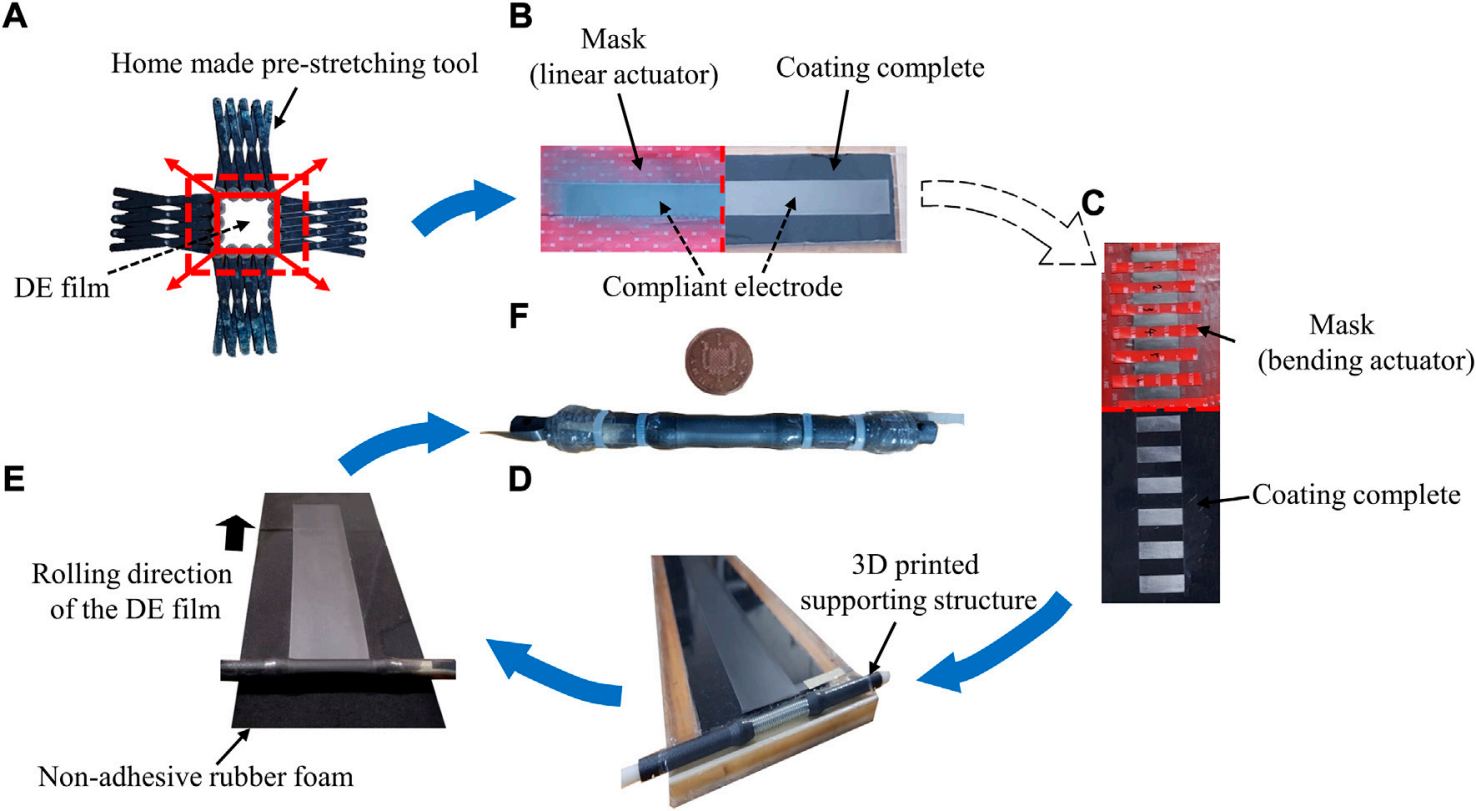
Fabrication procedure of the spring-roll DE actuator: **(A)** Pre-stretch of the DE; **(B)** Coating of the compliant electrode for linear-type DE actuator; **(C)** Coating of the compliant electrode for bending DE actuator; **(D)** Spring core accommodated in the beginning slot before rolling; **(E)** Rolling the spring core along the frame and wrapping DE films on it; **(F)** Fabricated sample beside a one penny coin.

Figure 8 shows the major steps that were followed during the fabrication of the spring-roll DE actuator. A homemade pre-stretching tool was designed and manufactured in this study to permit the stretching of the VHB 4905 elastomer film uniformly. A masking template was used to expose the required film area for the coating of the compliant electrode. The whole central area of the DE was exposed for the production of linear actuator, whilst only parts of the central area were exposed for the production of bending actuator. The fabricated DE actuators only weigh around 9 g.

### 3.2 Experimental arrangement

To determine the deformation of the DE actuator, a Digital Image Correlation (DIC) method, which is a non-invasive technique, was employed in this investigation to estimate the strain of the spring-roll DE actuator using captured digital images. A charge-coupled device (CCD) camera, type CM3-U3-13S2C-CS (supplied by Point Grey Company) was used to capture the deformed actuator images, which were subsequently imported and analyzed in a DIC processing software (GOM correlate). The actuator prototype was placed vertically, while the CCD camera was positioned horizontally using a clamping device. Electrical excitation of the actuator prototype was achieved using a dedicated power supply unit consisting of a portable high voltage DC converter, type XP Power FS50P-12 which was integrated with an adjustable power source, type QW-MS3010D. The power supply unit was calibrated in advance to provide the required voltage for the excitation of the actuator.

**FIGURE 9:**
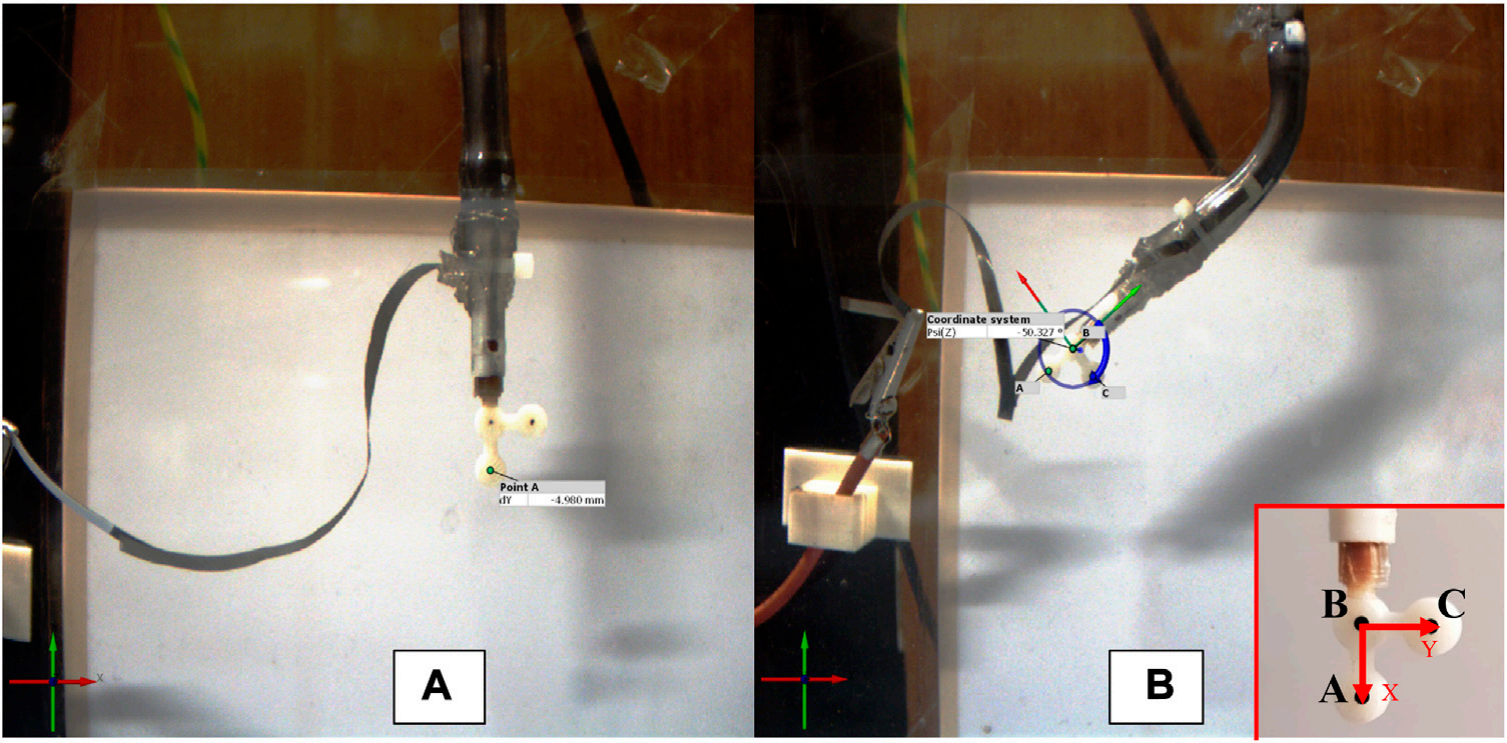
Marker recognition dot used in the deformation tests of linear-type DE actuator **(A)** and bending-type DE actuator **(B)**.

During the deformation assessment of the spring-roll DE actuators, a specially designed lightweight end tip (weight less than 1 g) was attached to the lower end of the actuator, as shown in the right bottom of Figure 9, on which three dots (A, B, C) markers were traced so that they could be identified by the software to estimate the displacement of actuator end tip during its activation stage while its top end is firmly fixed. These three dots are used to form a 2D coordinate system. The front surface of the end tip was positioned in the X-Y plane of deformation of the actuator, which was also parallel to the camera lens.

**FIGURE 10:**
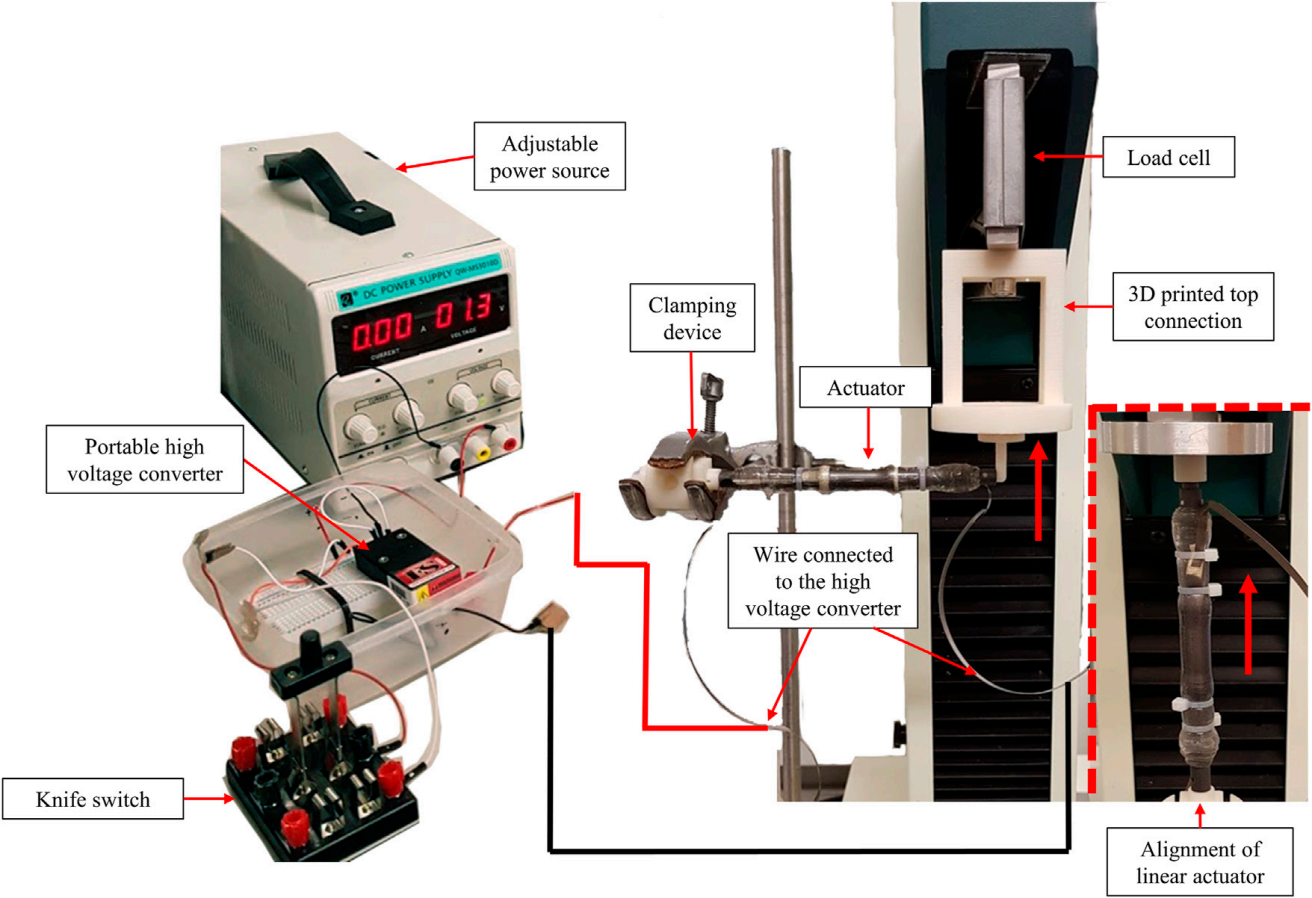
Force output test setup for bending-type spring-roll DE actuator (the alignment of the linear-type actuator as shown in the right bottom).

Experimental arrangements were made in which Tinus Olsen, type H5KS tensile machine was used to measure the force output of the spring-roll DE actuators as shown in Figure 10 for linear- and bending-type DE actuators. Special plastic connecting components were manufactured using a 3D printer, which was used to facilitate the alignment of the DE actuator (vertical for linear-type actuator and horizontal for bending-type actuator).

## 4 Results and discussions

### 4.1 Performance of the developed dielectric elastomer spring-roll actuator

**FIGURE 11:**
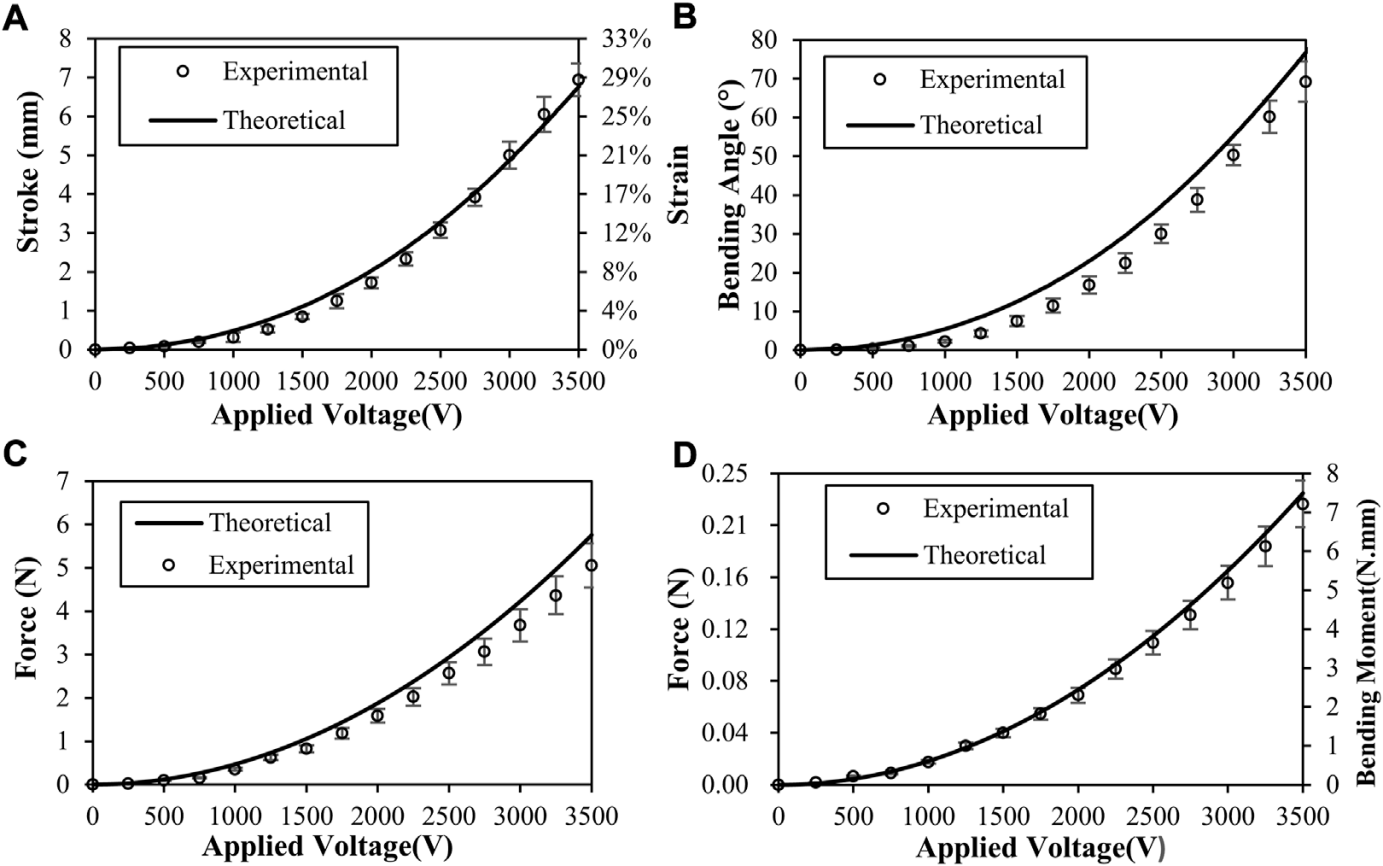
**(A)** Stroke/strain of linear-type spring-roll DE actuator *versus* applied voltage; **(B)** Angulation of bending-type spring-roll DE actuator *versus* applied voltage; **(C)** Output force of linear-type spring-roll DE actuator *versus* applied voltage; **(D)** Lateral blocking force/bending moment *versus* applied voltage for bending-type spring-roll DE actuator.

Experimentally, each test was repeated five times to validate the results whilst only the average values are reported. The variations of the linear stroke of the linear-type spring-roll DE actuator as a function of the applied voltage level are presented in Figure 11A. Accordingly, the DE roll was able to elongate to about 6.95 mm which is equivalent to an approximately 29% strain under the maximum applied voltage level of 3.5 kV.

Figure 11B shows that the maximum bending angle of the end tip of the spring-roll DE actuator could reach approximately 70°. On a few occasions, bending angles above 90° were observed when the applied voltage was increased beyond the level of 3.5 kV.

As presented in Figure 11C, a maximum blocking force magnitude of about 5.05 N was obtained for the linear-type actuator, which is about 12.3% lower than the theoretical force magnitude calculated as 5.76 N for the same voltage level.

The variation of the lateral blocking forces of the bending-type spring-roll DE actuator with the applied voltage is presented in Figure 11D. It can be seen that the blocking force of the bending-type DE actuator could reach a magnitude of approximately 0.22 N (bending moment about 7.2 N mm). Overall, it is observed that the standard deviation of the results grows with the increase of the excitation level of the DE actuator, which indicates that the performance of the actuator becomes less steady when the applied voltage was high.

### 4.2 Stability of the developed dielectric elastomer spring-roll actuator

**FIGURE 12:**
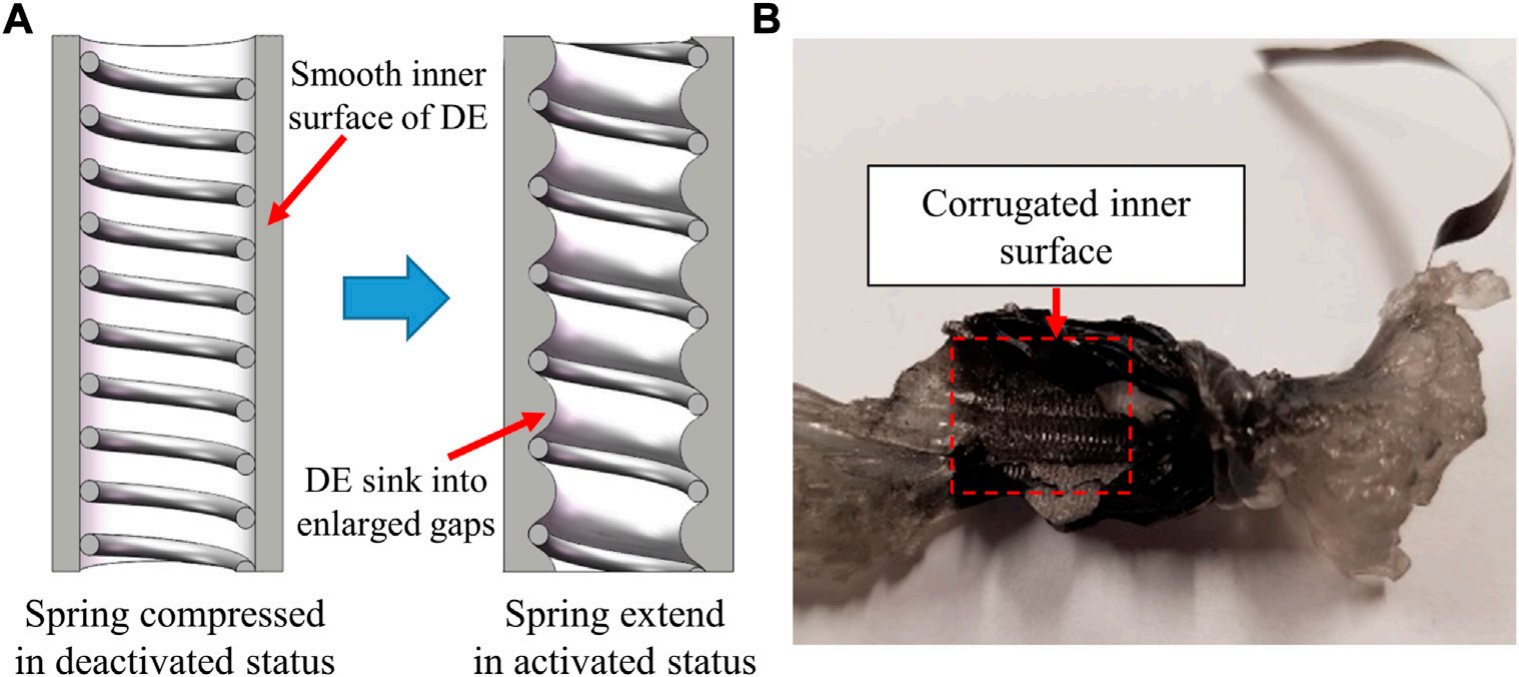
Inner surface variation of the spring-roll DE actuator: **(A)** Spring variation during the activation of the spring-roll DE actuator; **(B)** Corrugation of the surface of inner layers of the DE actuator.

As shown in Figure 11, the experimental deformation and force results of the DE actuators were seen to be in good qualitative agreement with the theoretical prediction. However, a deviation between the two sets of results still exists especially for elevated applied voltage levels. The undershooting of the experimental performance of the actuator may result from the corrugation of the inner surface of the DE. Ideally, the elastomer film should be rolled around the core spring of the spring-roll DE actuator, assuming that the spring has a smooth and flat surface. However, in actual practice situations, the support surface of the core spring cannot be maintained as a continuous plane surface, especially when the spring extends under the activated DE film action, where gaps between adjacent windings of the spring are enlarged and as a result, the surrounding elastomer film is expected to sink into these gaps and wrinkle in the radial direction as shown in Figure 12. This could be a major reason for the unstable performance of the prototypes when the excitation level was high.

Also, air bubbles, electrode connection, and minor film scars may cause a local electrical breaking down strength weakness or a non-uniform thickness distribution of the DE film across the width of the roll and the coated electrode. These issues are expected to either enhance the electric field locally, which may result in an early electrical breaking down failure, or cause mechanical damage to the DE film.

For future improvements, pad-printed electrodes could be utilized to mitigate this problem and improve the performance of the DE actuator (Ji et al., 2019). Furthermore, the procedures should be completed in a vacuum and dust-free environment to avoid any possible contamination.

### 4.3 Feasibility of the developed dielectric elastomer spring-roll actuator for minimally invasive surgery.

The output forces of the new actuator were found to be adequate to generate the angulation required by MIS tools with the comparison of actuators listed in Table 1. In addition, the developed DE spring-roll actuator utilizes a battery-operated miniature high voltage DC converter (type XP Power FS50P-12 as shown in Figure 10), which ensured the compatibility of such devices in MIS applications.

The DE material (VHB 4905) used in this study is a commercialized product, which remarkably lower the cost of the developed spring-roll DE actuator (the price of a single prototype could be lower than 16 CNY according to our trials). The weight of a single actuator is around 9 g. Moreover, the inside diameter of the actuator is 5.23 mm, which offers sufficient space for the passage of the guiding wires for the control of the tool’s end effector in addition to other possible functional structures.

Yet, the developed actuator has a few inevitable drawbacks. Due to the inherent viscoelastic behavior of the VHB material, the control dynamics of such devices were complicated. The low thickness of the VHB elastomer also adds difficulties to the fabrication of the DE actuator. Furthermore, the high voltage used in the DE actuator was considered to be dangerous by the general public, which impedes the development of DE actuators in the biomedical field. Despite that, haptic devices utilizing DE actuators have been developed by several research groups and the safety of such devices was assessed and deemed to be satisfactory for any contact with human subjects (Frediani et al., 2014; Boys et al., 2017; Pourazadi et al., 2017; Zhao et al., 2020; Guo et al., 2021).

Some researchers have proposed the utilization of non-compressive gel or soft non-conductive shielding membranes for an adequate separation between the high voltage and the users for compact DE haptic devices (Frediani et al., 2014; Boys et al., 2017), which could be a proper solution for soft DE actuators in medical use. In addition, the risk level of high voltage exposure mainly depends on the current intensity and charge duration exposed to the body, while the excitation of the small DE actuators is usually provided by a dedicated circuitry whose power output is only a few watts. And hence, some suggested that the safety concerns of small DE actuators could be mitigated, since the high voltage exposure of such devices could be avoided by limiting the current (usually less than 20 mA) and energy level of the electrical circuitry (Zhao et al., 2020). In this work, the maximum outputs of the DC converter (XP Power FS50P-12) are only 2 mA and 10 W, which is deemed to be safe even in medical scenarios.

Except for direct contact with the high voltage sources, the DE actuator is seen as a capacitive device under the excitation of the high voltage, which also rise concerns of high voltage discharge through its user. But research has indicated that the capacitance of the DE actuators is usually low, especially for compact DE devices (less than 300 pF per cm2 of a DE surface), which is considered to be not necessarily dangerous (Pourazadi et al., 2017). Therefore, with proper electrical shielding arrangements, the implementation of DE actuators for MIS tools is applicable. However, this paper is mainly focused on the optimization and performance evaluation of the DE actuator for MIS, while the detailed electrical shielding system and relative safety assessment will be carried out in future works.

## 5 Conclusion

This paper has introduced an investigation of dielectric elastomer (DE) spring-roll actuators which were aimed to improve the articulation of the current MIS surgical instruments. A new optimization process of design parameters was proposed to maximize the performance of the developed spring-roll DE actuators. In particular, the deformation behavior of the DE actuators and their force response were analyzed using theoretical and experimental methods, where the results from the two approaches were found to be in good agreement. The linear-type actuator could deliver a maximum strain of 29% and a blocking force up to 5.05 N, while the bending-type DE spring-roll actuator could deliver bending angles over 70° and a blocking force of up to 0.22 N (bending moment 7.2 N mm). Accordingly, the developed DE actuators were considered to be satisfactory for improving the function of the surgical instruments in MIS.

In addition, the spring-roll DE actuator is extremely lightweight and cheap to manufacture. Taking into account the unique soft features of such actuators, they can be used as a disposable devices in MIS applications such as an endoscope instrument, and other medical applications where a high level of dexterity is required. Accordingly, the developed optimization process could also provide design guidance for the spring-roll DE actuator in diverse scenarios by altering the corresponding design requirements.

Although the activation voltage of the spring-roll DE actuator is considered to be relatively high, which may raise safety concerns, practical measures were discussed to dampen such concerns and the corresponding electrical shielding system will be investigated in future works.

## Data Availability

The raw data supporting the conclusions of this article will be made available by the authors, without undue reservation.
